# Species distribution models for predicting the habitat suitability of Chinese fire‐bellied newt *Cynops orientalis* under climate change

**DOI:** 10.1002/ece3.7822

**Published:** 2021-06-27

**Authors:** Kun Guo, Sijia Yuan, Hao Wang, Jun Zhong, Yanqing Wu, Wan Chen, Chaochao Hu, Qing Chang

**Affiliations:** ^1^ Jiangsu Key Laboratory for Biodiversity and Biotechnology College of Life Sciences Nanjing Normal University Nanjing China; ^2^ College of Life and Environmental Science Wenzhou University Wenzhou China; ^3^ Nanjing Institute of Environmental Sciences Ministry of Environmental Protection Nanjing China; ^4^ College of Environment and Ecology Jiangsu Open University (The City Vocational College of Jiangsu) Nanjing China; ^5^ Analytical and Testing Center Nanjing Normal University Nanjing China

**Keywords:** *Cynops orientalis*, East Asia, habitat suitability, species distribution model

## Abstract

Climate change influences species geographical distribution and diversity pattern. The Chinese fire‐bellied newt (*Cynops orientalis*) is an endemic species distributed in East‐central China, which has been classified as near‐threatened species recently due to habitat destruction and degradation and illegal trade in the domestic and international pet markets. So far, little is known about the spatial distribution of the species. Based on bioclimatic data of the current and future climate projections, we modeled the change in suitable habitat for *C. orientalis* by ten algorithms, evaluated the importance of environmental factors in shaping their distribution, and identified distribution shifts under climate change scenarios. In this study, 46 records of *C. orientalis* from East China and 8 bioclimatic variables were used. Among the ten modeling algorithms, four (GAM, GBM, Maxent, and RF) were selected according to their predictive abilities. The current habitat suitability showed that *C. orientalis* had a relatively wide but fragmented distribution, and it encompassed 41,862 km^2^. The models suggested that precipitation of warmest quarter (bio18) and mean temperature of wettest quarter (bio6) had the highest contribution to the model. This study revealed that *C. orientalis* is sensitive to climate change, which will lead to a large range shift. The projected spatial and temporal pattern of range shifts for *C. orientalis* should provide a useful reference for implementing long‐term conservation and management strategies for amphibians in East China.

## Graphical abstract

Species distribution models were used to predict the habitat suitability of *Cynops orientalis*. Precipitation of warmest quarter and mean temperature of wettest quarter were the most critical factors shaping *C. orientalis* distribution. This species is sensitive to climate change, which will lead to the large range shift in future

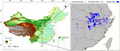

## 1 INTRODUCTION

Global climate change is occurring at an unprecedented rate, which may have significant influences on all levels of biodiversity from genes to ecosystems (Grünig et al., 2020). The Fifth Assessment Report (AR5) produced by the Intergovernmental Panel on Climate Change (IPCC) states that global warming is expected to continue with the average temperature of earth increasing by 0.3–4.5℃ by 2,100 compared with 1986–2005. Climate change has caused substantial geographical distribution changes in a wide variety of taxa from mammals, to birds, reptiles, and amphibians (Buckley et al., 2018; Kafash et al., 2018; Scridel et al., 2018; Subba et al., 2018). Many studies have shown that species respond differently to climate change, causing either expansion, shift, or contraction in the species ranges (Yousefi et al., 2020). For example, previous studies have demonstrated that in response to global warming, terrestrial species are shifting their distribution toward higher altitudes or latitudes (Zhang et al., 2018). Future climate change will probably result in large shifts in amphibian habitat suitability, thus determining substantial change in patterns of amphibian diversity in China (Duan et al., 2016). The largest extant amphibian species *Andrias davidianus* will lose more than two‐thirds of its suitable range in all scenarios of future climates tested based on SDM, and shift their range toward higher latitudes and altitudes (Zhang et al., 2020). However, the Chinese Skink *Eumeces chinensis* habitats are predicted to have no appreciable habitable range changes (Yang et al., 2020).

Species distribution modeling (SDM) is a common and effective method of assembling and presenting the spatial distributions of different taxa, including amphibians (Duan et al., 2016; Hu et al., 2016). In the past two decades, SDMs have emerged as one of the most effective techniques to investigate the impact of climate change in species habitat suitability (Araujo et al., 2019). With many different methods, tools, and protocols have been developed recently, various SDM methods have been used to evaluate the ecological requirements, ecological responses, and distribution areas (Guisan et al., 2017). SDMs provide useful information in terms of habitat suitability and help to find the climate conditions for future adaptation regarding conservation (Zhang et al., 2020).

SDMs are statistical models that use observed species distributional record data to infer species ecological requirements and map their habitat suitability (Austin, 2002). SDMs relate species presence records to mainly environmental factors to predict the potential distribution area (Pearson et al., 2004), which have been implemented in managing biological invasions, identifying and protecting critical habitats, selecting and translocating reserves, and so on (Thapa et al., 2018; Zhang et al., 2020). The most popular SDMs include MaxEnt, random forest, boosted regress trees, generalized additive models, and multivariate adaptive regression spines (García‐Callejas & Araújo, 2016).

**FIGURE 1:**
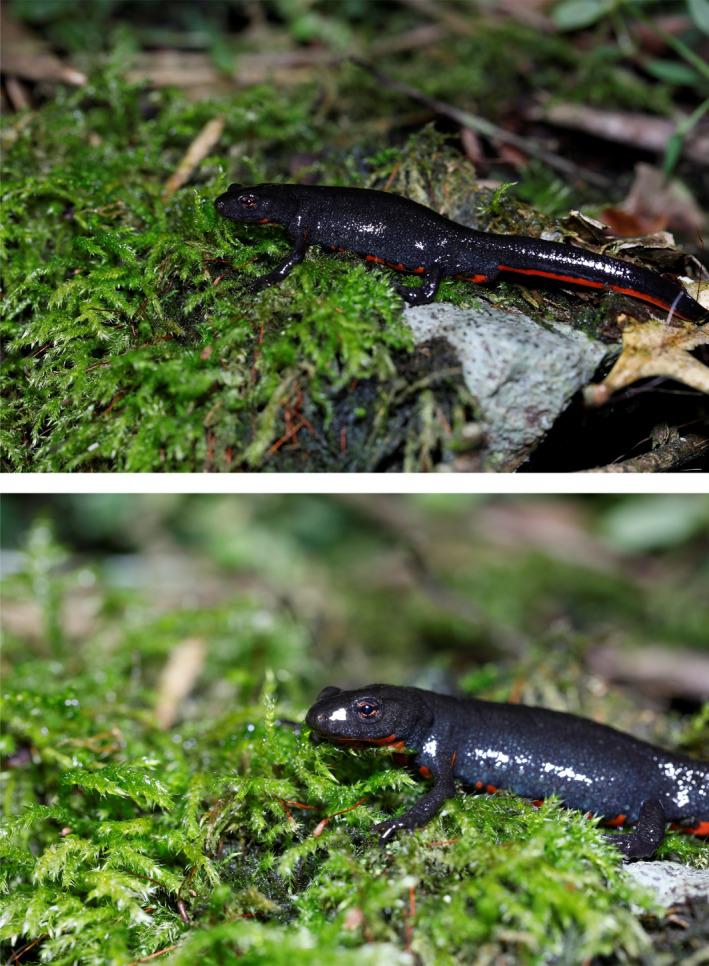
Chinese fire‐bellied newt from TaiZhou, Zhejiang Province, China. Photograph taken by Zhixiang Pan

Amphibians represent the most threatened vertebrate group in the world, for those population's declines many factors have been targeted as responsible, including by overexploitation, pollution, habitat degradation and destruction, diseases, invasive species, and climate change (Li et al., 2013). Among these, climate change is regarded as one of the most important drivers of amphibian extinction. Chinese fire‐bellied newt (*Cynops orientalis*) is an endemic species and distributed in East‐central China (Che & Wang, 2016), which is distributed on hilly lands or low mountain areas, and their habitats are ponds, streams, and wetlands found at low and middle elevations around slow‐moving bodies of water in China (Figure 1).

One crucial issue related to the ecological importance of *C. orientalis* is to determine how climate change will affect the spatial extent of their suitable habitat. To the best of our knowledge, no study has investigated potential impacts of future climate change in the habitat suitability for *C. orientalis* yet. To evaluate the properties of habitat distribution and environmental factors shaping suitability of habitat, we used ten models to predict distributions of *C. orientalis* in China using an extensive collection of georeferenced occurrence records and recent surveys. We structured our study under the hypothesis that climate change will result in the range contraction of *C. orientalis*, meanwhile pushing this species to higher altitudes and/or latitudes. The objectives of the present study include the following: (a) identifying the most important factors in model predicting, (b) determining the potential distribution of *C. orientalis* in current and future climate, and (c) comparing the future and current distribution patterns, and then quantifying the potential effect of climate changes. These findings will provide insight into *C. orientalis* habitat protection at the entire distribution range.

## 2 MATERIALS AND METHODS

### 2.1 Species occurrence records

**FIGURE 2:**
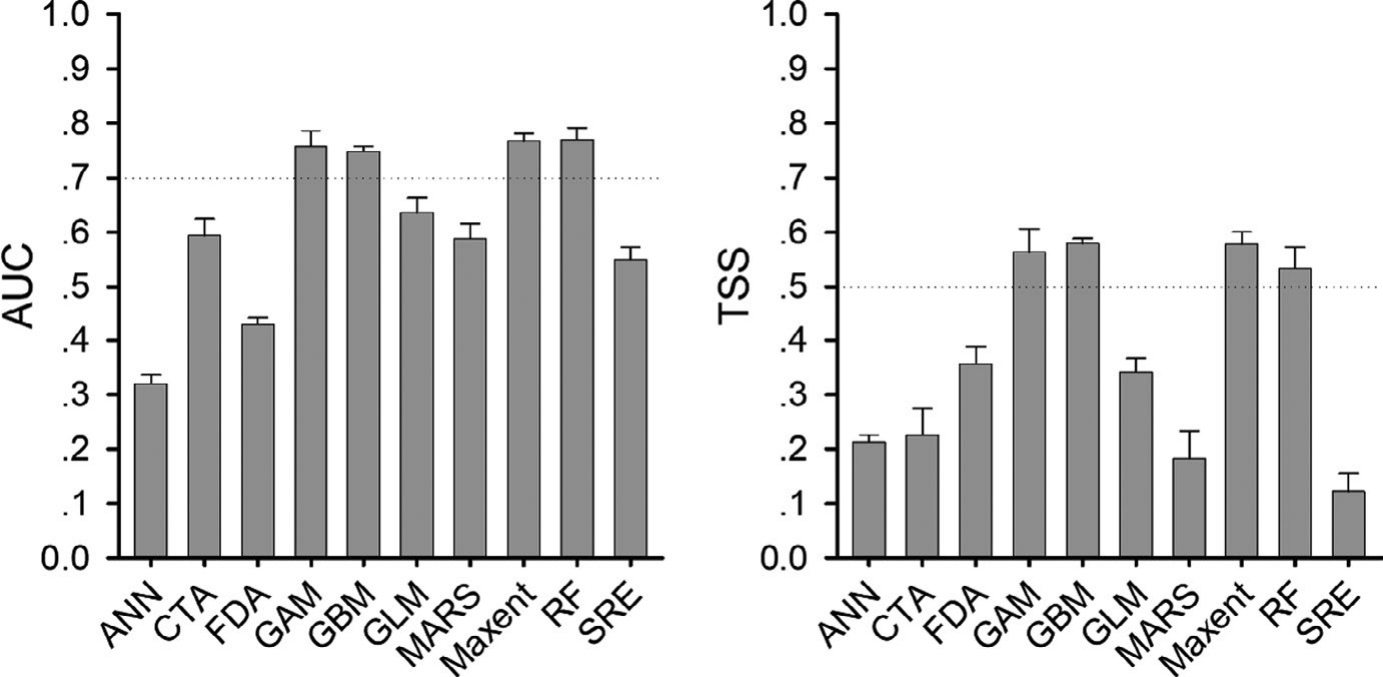
Predictive abilities (AUC and TSS) of ten modeling algorithms used to estimate habitat suitability for *Cynops orientalis*

Our study area is located in central and southern China, ranging from 110 to 124°E and 22 to 34°N (Figure 2). We determined the extent of the accessible area by considering the known geographical distribution of *Cynops orientalis* (Fei et al., 2012) and the distribution predicted by a previous SDM study focusing on this species (Chen, 2013). We assembled a database of recent georeferenced occurrence records of *C. orientalis* in China (Table S1). A total of 2,864 occurrence data were recorded based on field surveys, published work, and online resources like GBIF (http://www.gbif.org/). To minimize sampling bias effect in our dataset (Boria et al., 2014), we randomly selected only one record per each 2.5 × 2.5 arcmin grid cell (4.6 × 4.6 km at the equator), that is, the resolution of our environmental predictors (Boria et al., 2014). Records with obvious geocoding errors were discarded, and duplicate records were removed manually. Finally, we assembled a database of 46 spatially georeferenced occurrence records for model calibration, covering through central and southern China, which conformed to minimum required number for accurate projection (Van Proosdij et al., 2016). Although we acknowledge that the sample size is small and that the ultimate strength of the SDM inference may be affected as a result, previous studies revealed that SDMs based on small sample size can also provide useful predictions (Hernandez et al., 2006; Pearson et al., 2006).

### 2.2 Environmental predictors

We downloaded the standard 19 bioclimatic variables and elevation with a resolution of 2.5 arcmin from WorldClim version 2.1 (available at https://www.worldclim.org), which are the average for the years 1970–2000 (Fick & Hijmans, 2017). With potential direct or indirect relationships on the life history of amphibians, these bioclimatic variables have been widely used in SDMs (Malekoutian et al., 2020; Sangermano et al., 2015). For the purpose of avoiding the effect of redundancy, we filtered the variables among 19 Bioclimatic variables by pairwise Pearson's correlation coefficients (*r*), thereby selecting the restrictive variables for *C. orientalis* with the value of |*r*| to be less than 0.70 (Dai et al., 2018). We also downloaded the future climate projections with the same spatial resolution of current period data from WorldClim for four periods, 2030 (average for 2021–2040), 2050 (average for 2041–2060), 2070 (average for 2061–2080), and 2090 (average for 2081–2100), under two shared socio‐economic pathway (SSP) scenarios (i.e., SSP245 and SSP585). To reduce uncertainties among different GCMs, we averaged projections of six GCMs as future climates to predict future habitat suitability of *C. orientalis*.

### 2.3 Modeling procedure

To develop an accurate projection for *C. orientalis*, we used an ensemble modeling approach of ten algorithms in biomod2 package (See the section of Abbreviations) (Thuiller et al., 2019). We sampled 10,000 pseudo‐absences from the study area to obtain pseudo‐absences or background records that are required for several algorithms (Guisan et al., 2017). We used a fivefold cross‐validation approach to run the models, in which 80% of data (presences and pseudo‐absences) were randomly chosen for model training and the last were remained for model test (Guisan et al., 2017; Thuiller et al., 2019). We used two criterion parameters, true skill statistics (TSS) and area under the receiver operating characteristic curve (AUC) to evaluate predictive performances of the algorithms (Allouche et al., 2006). The algorithms with TSS ≥0.50 and AUC ≥0.70 were selected to estimate relative contributions of predictor variables in determining the distribution by a randomization method (Guisan et al., 2017) and to project habitat suitability of *C. orientalis* under current and future climates (Gallien et al., 2012).

The committee averaging approach taking into account averages of all model predictions with the same weight was used to reduce the algorithms bias effect in the single predictions (Guisan et al., 2017; Thuiller et al., 2019). For a better interpretation of distribution changes under present and future climates, we converted potential distribution projections into binary maps (suitable/unsuitable) by maximizing the TSS value (Guisan et al., 2017; Liu et al., 2013).

We counted the number of raster cells (2.5 × 2.5 arcmin grid cell) classified as the loss areas, stable areas, and gain areas to estimate changes in range size by comparing suitable habitats under future and current climate conditions (Thuiller et al., 2019). The elevation of suitable raster cells under current and future climate scenarios was extracted to evaluate changes in altitudinal distribution of *C. orientalis* with one‐way ANOVA and Tukey's post hoc test at the significance level of 0.05.

## 3 RESULTS

### 3.1 Model performances and contributions of predictor variables

**FIGURE 3:**
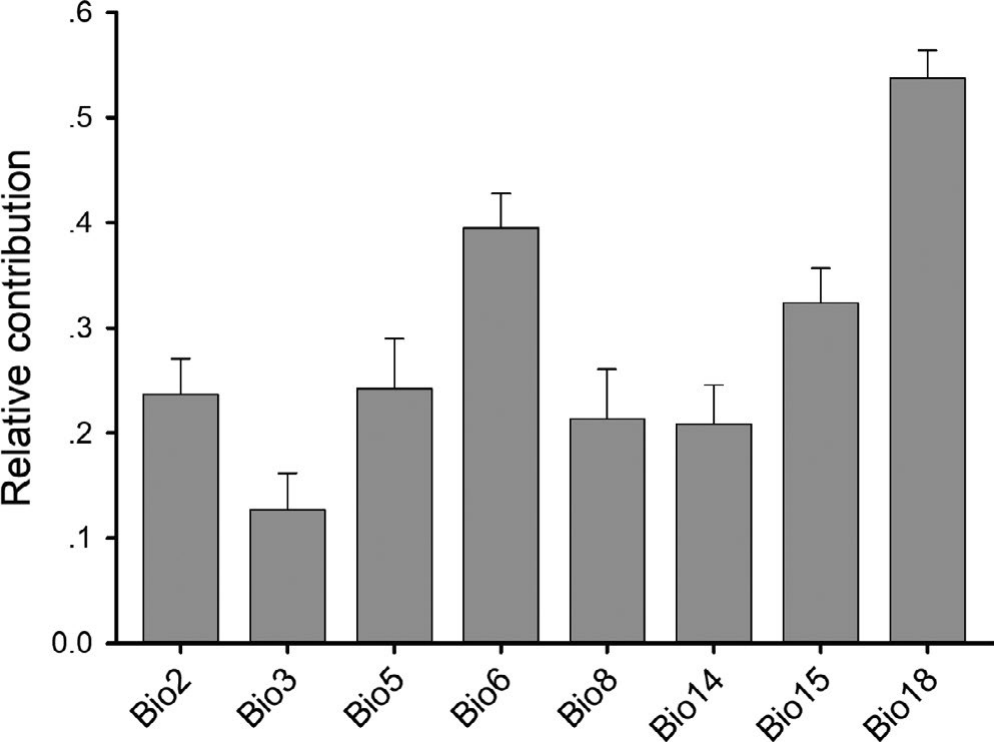
Relative contributions of the eight selected predictor variables in the ensemble model of habitat suitability for *Cynops orientalis*

According to the results of pairwise Pearson's correlation coefficients (Figure S1), eight predictor variables (mean diurnal range, bio2; isothermality, bio3; temperature seasonality, bio4; max temperature of warmest month, bio5; min temperature of coldest month, bio6; mean temperature of wettest quarter, bio8; precipitation of driest month, bio14; precipitation seasonality, bio15; and precipitation of warmest quarter, bio18) were selected to develop SDMs for *C. orientalis* (Figure S1). Among the ten modeling algorithms, four (GAM, GBM, Maxent, and RF) had clearly better predictive performances than the others (Figure 2). On the basic values of relative contribution, bio18 was the most important variable to limit the habitat suitability of *C. orientalis*, followed by bio6 (Figure 3, Figure S3).

### 3.2 Habitat suitability under present and future climates

**FIGURE 4:**
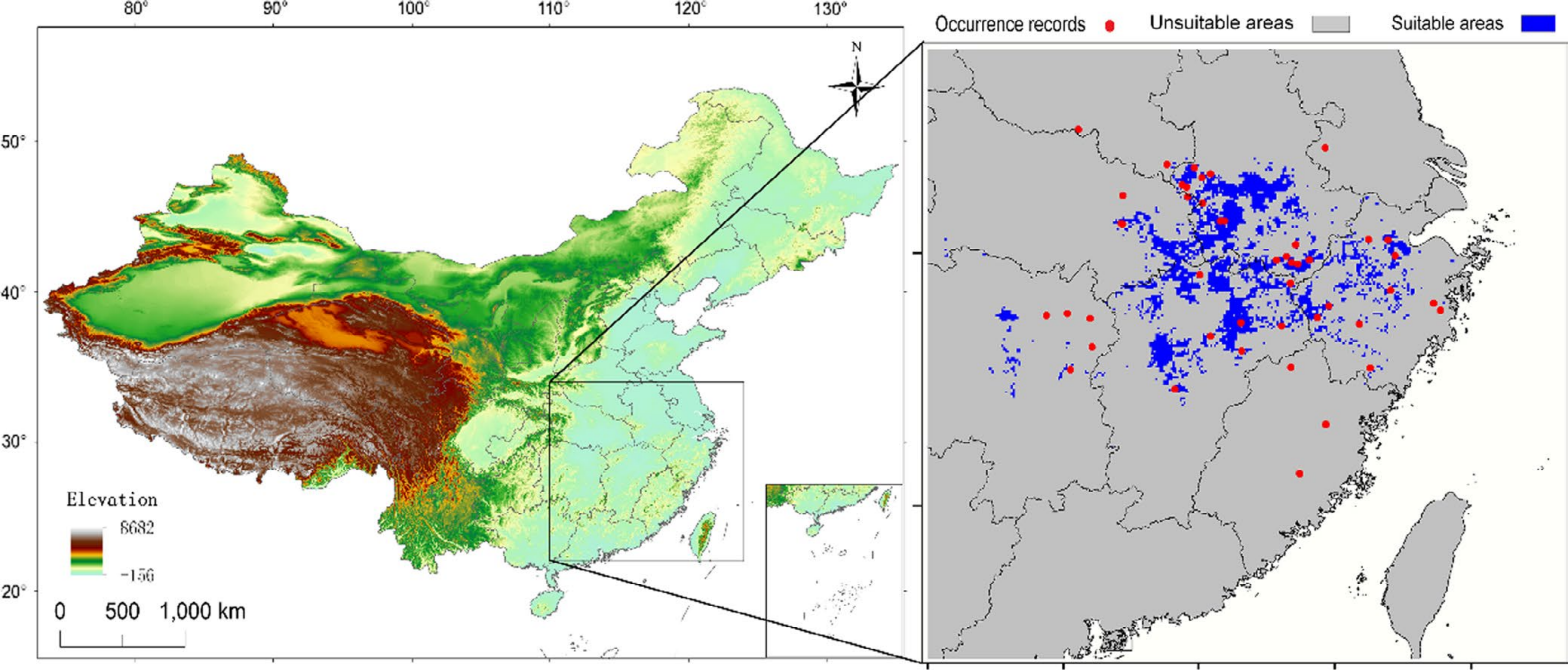
Binary output of habitat suitability for *Cynops orientalis* under current climate conditions. Blue color indicates suitable area, and gray color represents unsuitable range. Black pane shows the study area

**FIGURE 5:**
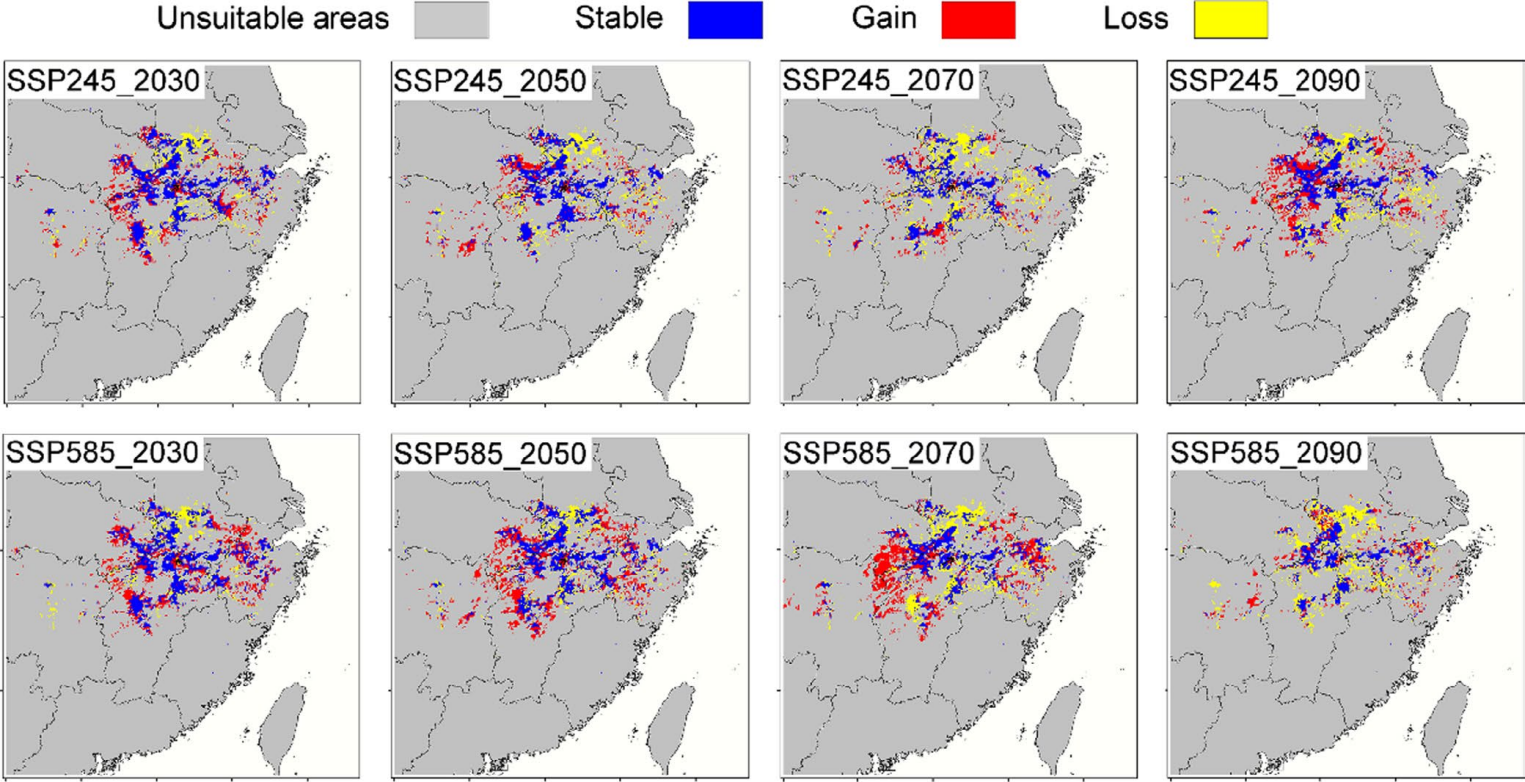
The current and future suitable habitats of *Cynops orientalis*. Blue areas are currently suitable habitats that may still be suitable in the future. Red areas are suitable habitats added in the future. Black areas are currently suitable habitats that become unsuitable in the future

**FIGURE 6:**
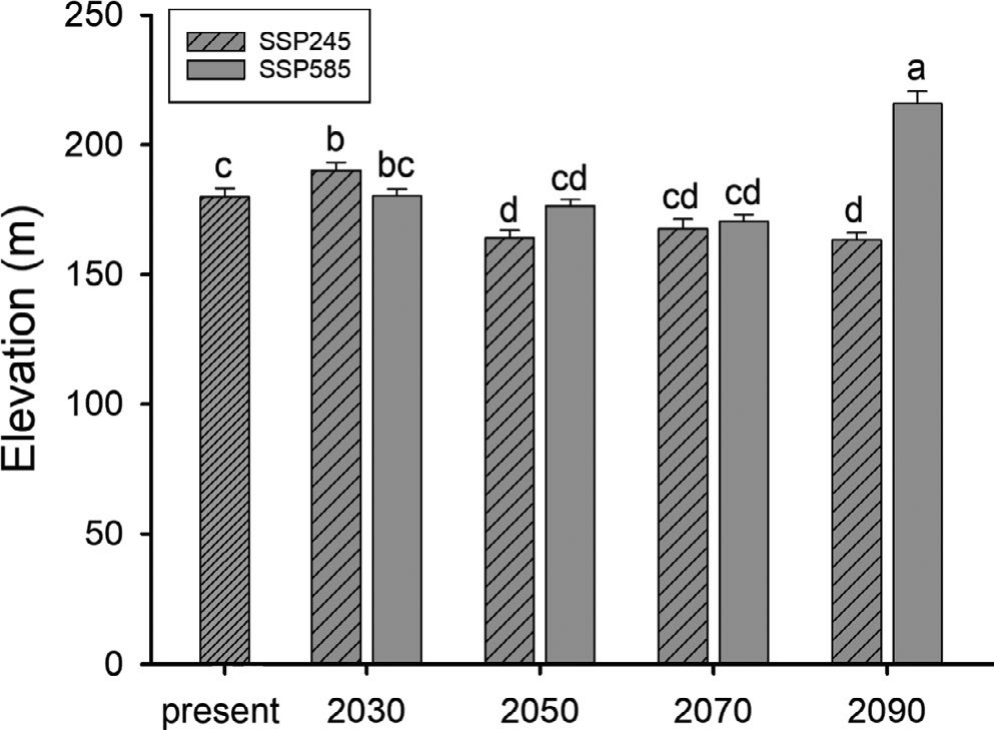
Mean (±*SE*) elevation values within suitable raster cells under present and future climate conditions

The result of projection suggested that the suitable habitat for this species is mainly located in Eastern China, including Hunan, Jiangxi, Zhejiang, Anhui, and Hubei provinces (Figure 4). Besides, small areas in Jiangsu and Fujian provinces also have suitable habitat. The result of range changes indicated that six of the eight models showed range expansion under future climate conditions (Table 1), and the gain areas would appear at the south of suitable habitat (Figure 5). The projections of habitat suitability for *C. orientalis* under future climate condition varied depending on the SSPs used. We especially found that the distribution range under SSP245 would expand by 18.07% in the 2030, while that of under SSP585 would expand by 29.75%. On the contrary, the range under SSP585 would contract by 27.38% in the 2090, while that of under SSP245 would expand by 19.36% (Table 1). The elevation of habitat suitability under present climate condition (179.85 ± 3.36 m) was found to be significantly greater than those habitat suitability under future climate condition (*F*_8, 35,626_ = 22.24, *p* < .001), except for that of the 2090 under SSP585 and 2030 under SSP245 (Figure 6).

**TABLE 1:** Range size change in *Cynops orientalis* in 2030, 2050, 2070, and 2090 under two SSPs

Years	Shared socio‐economic pathways	Range change (%)
2030	SSP245	18.07
	SSP585	29.75
2050	SSP245	0.03
	SSP585	39.99
2070	SSP245	–25.52
	SSP585	24.42
2090	SSP245	19.36
	SSP585	–27.38

## 4 DISCUSSION

In this study, we performed a detailed analysis on the suitable habitat of the Chinese fire‐bellied newt *C. orientalis* under current and future climate conditions, which will function as an important step in formulating a feasible strategy for their conservation. Our model indicated that the suitable habitat area encompassed ca. 41,862 km^2^ for the species under current climate conditions and the center area was in southwest Anhui and north Jiangxi provinces. With good predictive accuracy, this study revealed that the *C. orientalis* has a relatively wide distribution, but the suitable habitats are discontinuous, showing the characteristics of a fragmented distribution. Our results show that *C. orientalis* is very sensitive to climate change, which will lead to the large range shift (loss and/or gain) of suitable habitats (Figures 5, 6, and Figure S2).

Among the ten variables adopted in the model, bio18 and bio6 made the most contributions to the distribution model for *C. orientalis* compared with other variables, indicating that these two factors play important roles in its distribution. Climate is a more important driving factor than elevation, suggesting that microclimates in the East China make for a powerful driver of *C. orientalis* ecological niche and distribution. Variables related to extreme environmental conditions (precipitation of warmest quarter and the mean temperature of wettest quarter) also emerged as important in explaining the distribution of *C. orientalis*. Precipitation is closely related to the amount of water in ponds, streams, and slow‐moving wetlands as well (wet conditions). Temperature plays an important role in determining species' distributions, and evaluating the influence of climatic variables across a large geographic area to provide information about suitable habitat for a given species. The suitable temperature ranges for *C. orientalis* were 15–25℃ (Lu et al., 2017). This suggests a physiological limit to the distribution of *C. orientalis*.

With global warming, some species will migrate to high latitude or high elevation, while other species may adapt to these changes physiologically or phenologically (Li et al., 2013; Zhang et al., 2020). In this study, future projection indicates that the suitable range of *C. orientalis* will not shift toward higher altitudes and latitudes. Habitat suitability under current and future scenarios showed the large range shift (loss and/or gain), with more habitat suitability toward the south parts of study area and some scattered locations. These findings are not consistent with our initial working hypotheses. Climate change‐induced altitudinal and/or latitudinal range shifts have been observed in a number of organisms, which reveal that amphibians prefer the wet conditions more available in the northeast (Chen et al., 2011; Poloczanska et al., 2013). Amphibians are experiencing global declines, and thus, a number of SDMs have been built to estimate their habitat suitability under future climate conditions (Duan et al., 2016; Popescu et al., 2013; Zhang et al., 2020). In fact, evidence that climate change is directly contributing to amphibian declines is weak, partly because researchers have not often ruled out alternative hypotheses, such as chytrid fungus or climate–fungus interactions (Li et al., 2013). We should consider that species dispersal capability is a critical limiting factor influencing species distribution and SDM projections can be largely divergent depending on different assumptions regarding dispersal abilities (Guisan et al., 2017; Li et al., 2013). The suitable area of *C. orientalis* in future was not higher than current habitat. In the present SDM study, we adopted an unlimited dispersal ability assumption and our SDM projections suggest that in addition to considerable range shift, *C. orientalis* will expand its range in some provinces. It is unclear whether *C. orientalis* has the ability to keep pace with climate change‐induced range shifts and colonize new areas.

It is worth noting that climate change will also determine substantial environmental changes in the microclimatic zones. East China is a mosaic of mountains lower than 2000 m and characterized by a relatively mild climate, potentially hosting microclimatic zones capable of supporting a variety of habitats in relative stability (Ju et al., 2007; Qian & Ricklefs, 2000). The mountains might intercept moisture and heat, transported by monsoons from the ocean, providing relative climatic and ecological stability. Temperature data from microhabitats and macrohabitats in primary rainforests show that microhabitats can reduce mean temperature and the duration of extreme temperature exposure; thus, microhabitats have the potential to buffer species from climate change (Scheffers et al., 2014). Microclimatic zones may have mitigated demographic stresses and also have been available for East Asian species (Li et al., 2009). While the effects of climate change are largely studied in surface habitats, the impacts on microclimatic habitats are still poorly explored (Mammola et al., 2019). The temperature increment in these transitional microclimatic habitats is expected to parallel the external one almost synchronically (Mammola et al., 2019). Furthermore, climate change is expected to determine drops in relative humidity and even desiccation of subterranean habitats (Mammola et al., 2019). This species occurs only in very small locations, and based on the loss‐gain maps, these species will tend to gain more than they lose under climate change. Local disasters, such as diseases, drought and land transformation by human activities, can easily cause extinction (Li et al., 2013; Shu et al., 2013). Climate change‐induced range contraction will sum up to other threats that are currently severely affecting this species, from commercial overexploitation for human consumption to habitat degradation and disruption (Turvey et al., 2018). In view of our results, we strongly recommend that future adaptive management strategies should take into consideration the potential impacts of climate change in *C. orientalis*.

As a result, increased use of illegal trade in the domestic and international pet markets may lead to extinction that occurs despite the presence of suitable habitats now and in the future. Climatic change will not result in a decrease in the availability of suitable habitats. The present study suggests that *C. orientalis* is vulnerable to climate change, which will lead to the large range shift (loss and/or gain) of suitable habitats under future climatic conditions. However, large amounts of currently suitable habitat may disappear because of land‐use change and human use for illegal trade pet market purposes. This study will provide a useful reference for implementing long‐term conservation and management strategies for amphibians.

## CONFLICT OF INTEREST

The authors declare that they have no conflict of interest.

## AUTHOR CONTRIBUTIONS

**Kun Guo:** Formal analysis (lead); investigation (lead); methodology (lead); software (lead). **Sijia Yuan:** Investigation (lead); validation (equal). **Hao Wang:** Data curation (equal); formal analysis (equal); resources (equal). **Jun Zhong:** Formal analysis (equal); software (lead). **Yanqing Wu:** Conceptualization (equal); data curation (equal); investigation (lead); methodology (lead). **Wan Chen:** Funding acquisition (lead); validation (lead); writing–original draft (lead). **Chaochao Hu:** Conceptualization (lead); data curation (lead); resources (lead); validation (lead); writing–original draft (lead). **Qing Chang:** Conceptualization (equal); validation (equal).

## Supporting information

Supplementary Material

## Data Availability

Environmental data can be obtained from CHELSA (http://chelsaclimate.org) and WorldClim (http://www.world clim.org). Sampling site information is provided in Table S1.
