# Supplementary material for: Species distribution models for predicting the habitat suitability of Chinese fire‐bellied newt Cynops orientalis under climate change
Source: Ecol Evol. 2021 Jun 27;11(15):10147–54. doi: 10.1002/ece3.7822 (PMC8328465; doi:10.1002/ece3.7822)
Supplement: Supplementary file 1 — Supplementary Material [file ECE3-11-10147-s001.docx]

Appendix

Figure S1: Relative contributions of the 19 selected predictor variables in the ensemble model of habitat suitability for *C. orientalis.*


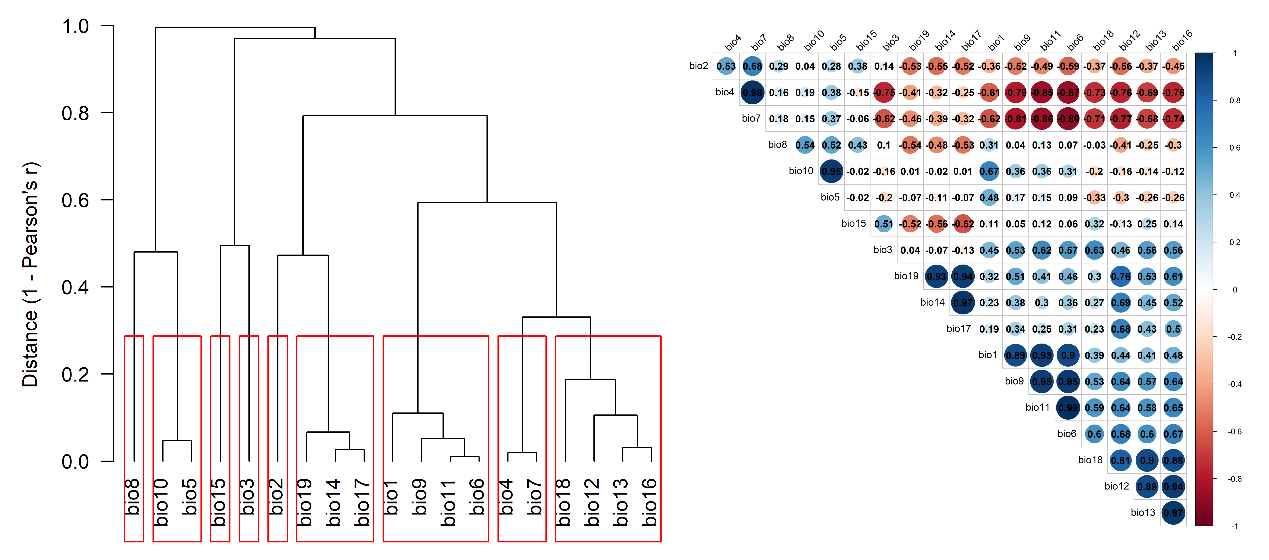


Figure S2: Frequency distribution of altitude for *C. orientalis.*


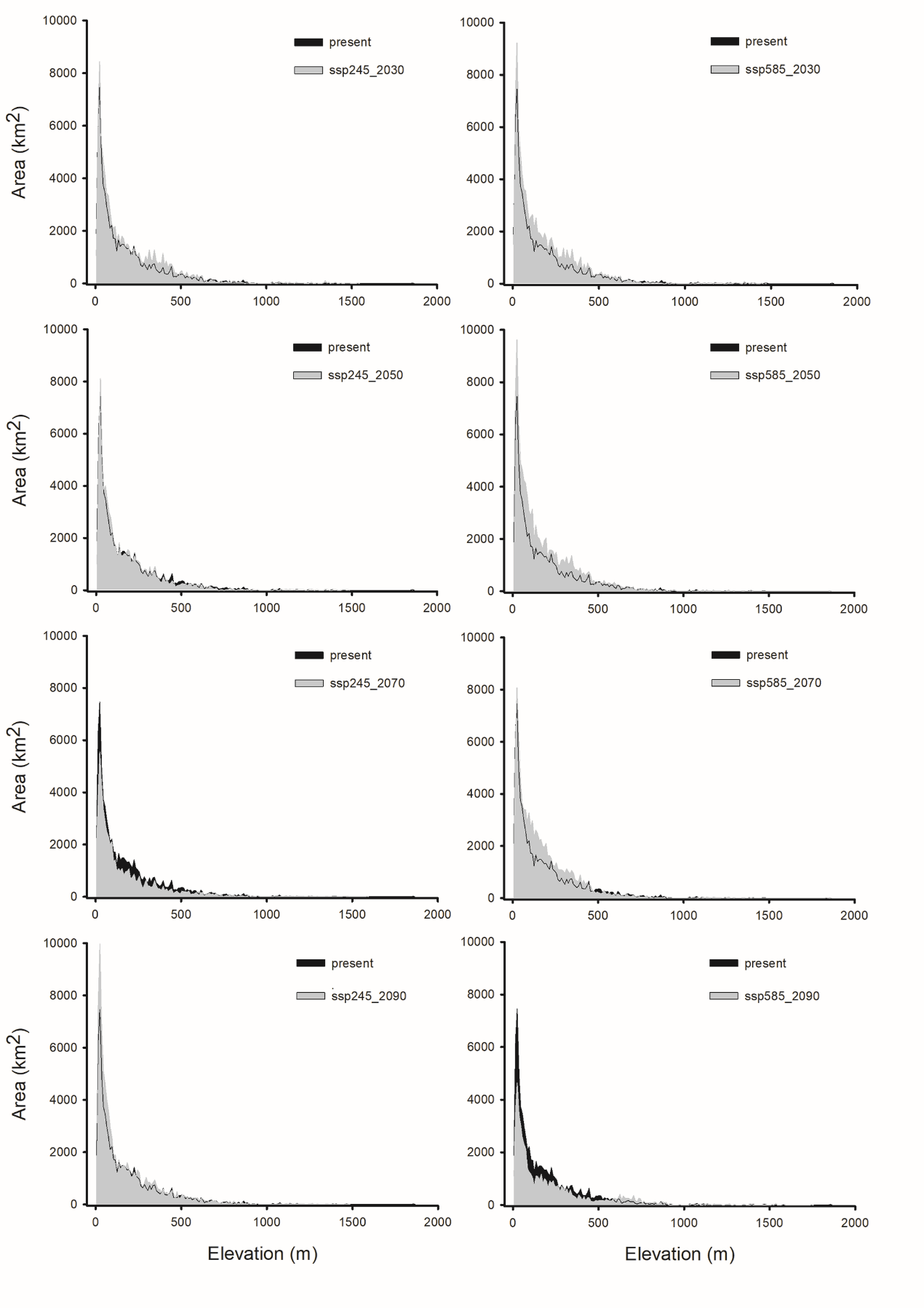


Figure S3: Response curves of predicted occurrence probability of *C. orientalis* against eight predictor variables.


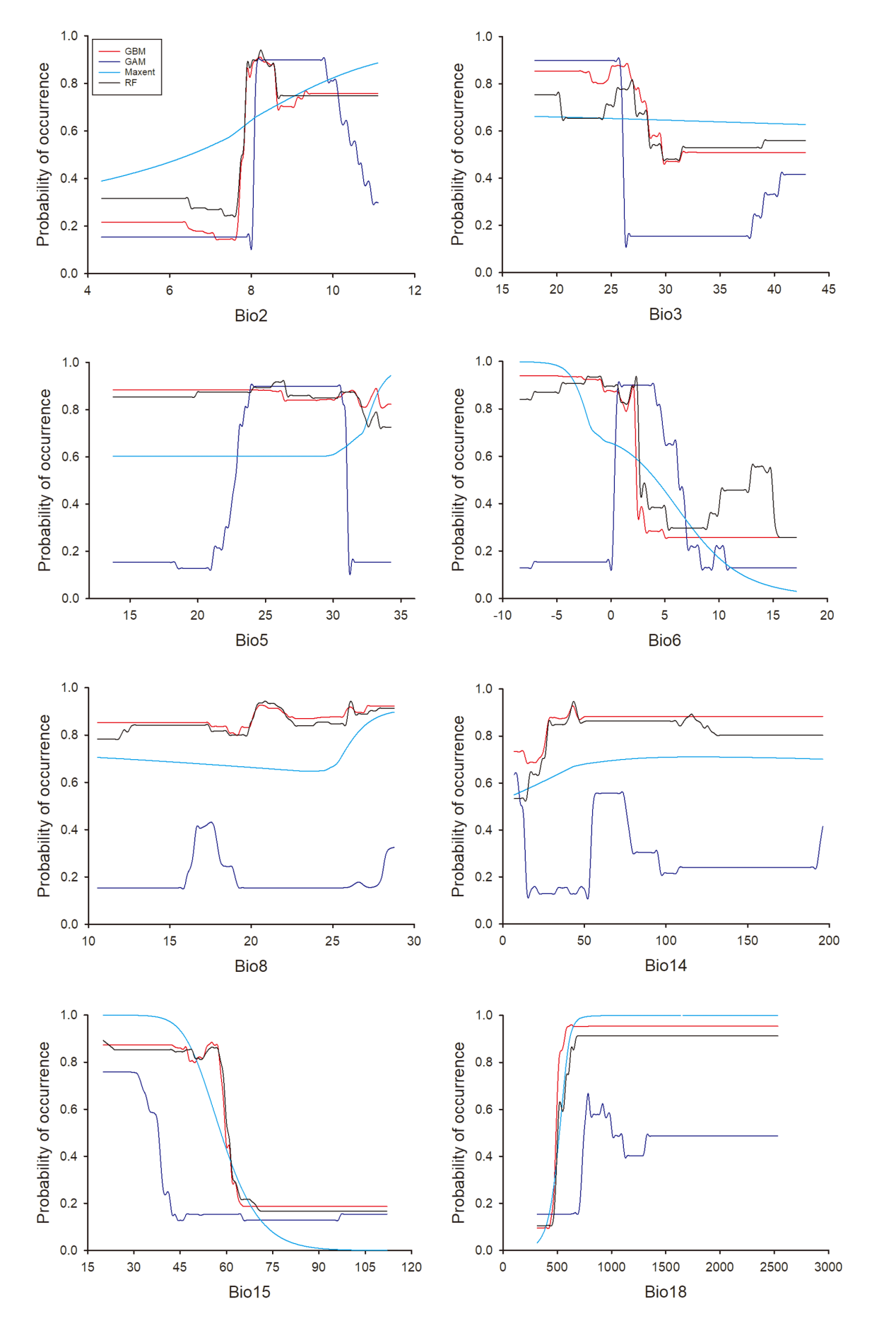


Table S1 Detailed distributional records of *Cynops orientalis* in the present study.

|  | Order | longitude | latitude |
| --- | --- | --- | --- |
| *Cynops orientalis* | 1 | 115.7550222 | 31.10225556 |
| *Cynops orientalis* | 2 | 115.6407806 | 31.34253611 |
| *Cynops orientalis* | 3 | 115.7314972 | 31.29023333 |
| *Cynops orientalis* | 4 | 116.075 | 31.48211389 |
| *Cynops orientalis* | 5 | 118.0449333 | 29.81480278 |
| *Cynops orientalis* | 6 | 116.0801611 | 30.98108056 |
| *Cynops orientalis* | 7 | 116.56848 | 30.62742 |
| *Cynops orientalis* | 8 | 118.221833 | 25.660833 |
| *Cynops orientalis* | 9 | 114.265604 | 30.583645 |
| *Cynops orientalis* | 10 | 118.7778 | 32.0617 |
| *Cynops orientalis* | 11 | 116.92628 | 28.63111 |
| *Cynops orientalis* | 12 | 117.8275 | 28.56035 |
| *Cynops orientalis* | 13 | 118.01806 | 29.40083 |
| *Cynops orientalis* | 14 | 120.32351 | 29.94275 |
| *Cynops orientalis* | 15 | 120.169 | 30.255 |
| *Cynops orientalis* | 16 | 120.2253 | 29.2678 |
| *Cynops orientalis* | 17 | 119.773 | 27.737 |
| *Cynops orientalis* | 18 | 118.796 | 26.63 |
| *Cynops orientalis* | 19 | 121.174 | 29.009 |
| *Cynops orientalis* | 20 | 119.533 | 28.604 |
| *Cynops orientalis* | 21 | 113.0808 | 28.81172 |
| *Cynops orientalis* | 22 | 113.1475 | 27.71063 |
| *Cynops orientalis* | 23 | 113.5749 | 28.70981 |
| *Cynops orientalis* | 24 | 113.6214 | 28.14957 |
| *Cynops orientalis* | 25 | 114.2919 | 30.56751 |
| *Cynops orientalis* | 26 | 116.2591 | 28.36449 |
| *Cynops orientalis* | 27 | 117.707 | 29.85415 |
| *Cynops orientalis* | 28 | 117.9302 | 29.93312 |
| *Cynops orientalis* | 29 | 118.0268 | 27.75603 |
| *Cynops orientalis* | 30 | 118.1758 | 29.78119 |
| *Cynops orientalis* | 31 | 118.4339 | 29.86577 |
| *Cynops orientalis* | 32 | 118.6159 | 28.73531 |
| *Cynops orientalis* | 33 | 118.8692 | 28.95845 |
| *Cynops orientalis* | 34 | 119.7348 | 30.26118 |
| *Cynops orientalis* | 35 | 115.4723 | 27.32133 |
| *Cynops orientalis* | 36 | 114.2974 | 31.13217 |
| *Cynops orientalis* | 37 | 115.298 | 31.74049 |
| *Cynops orientalis* | 38 | 113.3236 | 32.43528 |
| *Cynops orientalis* | 39 | 116.0252 | 29.56568 |
| *Cynops orientalis* | 40 | 112.6217 | 28.77522 |
| *Cynops orientalis* | 41 | 116.4772 | 30.6283 |
| *Cynops orientalis* | 42 | 115.9039 | 31.6762 |
| *Cynops orientalis* | 43 | 116.9431 | 28.07498 |
| *Cynops orientalis* | 44 | 118.1346 | 30.16064 |
| *Cynops orientalis* | 45 | 121.3252 | 28.86709 |
| *Cynops orientalis* | 46 | 116.2606 | 31.55748 |
